# Potentially toxic element (PTE) levels in maize, soil, and irrigation water and health risks through maize consumption in northern Ningxia, China

**DOI:** 10.1186/s12889-020-09845-5

**Published:** 2020-11-16

**Authors:** Ping Liu, Yahong Zhang, Ningchuan Feng, Meilin Zhu, Juncang Tian

**Affiliations:** 1 School of Civil and Hydraulic Engineering, Ningxia University, Yinchuan, 750021 China; 2 School of Physics and Electronic-Electrical Engineering, Ningxia University, Yinchuan, 750021 China; 3 College of Pharmacy, Ningxia Medical University, Yinchuan, 750004 China; 4 College of Basic Medical Sciences, Ningxia Medical University, Yinchuan, 750004 China

**Keywords:** Potentially toxic element, Bioaccumulation, The Yellow River, Potential health risk, Monte Carlo simulation

## Abstract

**Background:**

Industrial and agricultural activities result in elevated levels of potentially toxic elements (PTEs) in the local environment. PTEs can enter the human body through the food chain and pose severe health risks to inhabitants. In this study, PTE levels in maize, soil, and irrigation water were detected, and health risks through maize consumption were evaluated.

**Methods:**

Maize, soil, and irrigation water samples were collected in northern Ningxia, China. Inductively coupled plasma-optical emission spectrometry was applied to determine the contents of six PTEs. Bioaccumulation factor was used to reflect the transfer potential of a metal from soil to maize. Health risks associated with maize consumption were assessed by deterministic and probabilistic estimation. Sensitivity analysis was performed to determine variables that pose the greatest effect on health risk results.

**Results:**

The levels of Pb and Cr in maize exceeded the standards, while the PTE levels in soil and irrigation water did not exceed the corresponding standards. The bioaccumulation factor values of the six PTEs in maize were all lower than 1 and followed the order of Cd > Zn = As > Cr > Cu > Pb. The hazard index (0.0986) was far less than 1 for all inhabitants implying no obvious non-carcinogenic risk. The carcinogenic risk value was 3.261 × 10^− 5^, which was lower than the maximum acceptable level of 1 × 10^− 4^ suggested by United States Environmental Protection Agency (USEPA). Females were at greater risk than males, and the age group of below 20 years had the greater risk among all the groups evaluated. Approximately 0.62% of inhabitants exceeded the level for non-carcinogenic risk, while 8.23% exceeded the level for carcinogenic risk. The As concentration and daily intake of maize contributed 35.8, and 29.4% for non-carcinogenic risk results as well as 61.0 and 18.5% for carcinogenic risk results.

**Conclusions:**

Maize was contaminated by Pb and Cr, whereas the associated soil and irrigation water were not contaminated by PTEs. Inhabitants would not suffer obvious harmful health risks through maize consumption. Arsenic level and daily intake of maize were the most sensitive factors that impact health risks.

**Supplementary Information:**

The online version contains supplementary material available at 10.1186/s12889-020-09845-5.

## Background

Environmental contamination has become a serious problem in developing countries as a result of rapidly increasing population growth, industrial and commercial development, and accelerated urbanization [1]. PTEs are an important type of contaminant that can accumulate in the environment from sources such as mining, pesticides, and chemical fertilizers [2]. In China, the environment is more heavily contaminated by PTEs in regions with higher degrees of industrialization. Industrial wastes such as waste water, waste residue, and flue gas can affect the surrounding agricultural land, water, and air. In a previous study, the PTE concentration in 29.4% of soil samples collected from 2523 industrial parks exceeded the standard for soil environmental quality [3]. Due to the toxicity and persistence of toxic elements, PTEs pollution in soil were more harmful than other soil pollutants [4–6]. For example, cadmium (Cd) exposure can cause adverse health effects, including damage to the lung, liver, testicles, brain, bone, and blood system along with cancer; Cd can accumulate for 10 to 20 years in the human body and is considered one of the most toxic PTEs [7–9]. The main toxic effects of lead (Pb) are neurological effects, especially intelligence quotient (I.Q.) deficits [10]. In children, Pb may cause behavioral disturbances along with learning and concentration difficulties [11]. Although arsenic (As) is a metalloid, it is considered as a metal in many studies because it behaves similar to a PTE in many aspects. When As enters the body, it is primarily deposited in the hair, bones, and other organs and can lead to disorders of the respiratory, nervous, and circulation systems along with cancer [12, 13]. Although some PTEs including copper (Cu), zinc (Zn), and chromium (Cr) are considered essential micronutrients at low concentrations [14, 15], they can pose non-carcinogenic hazardous effects on human health when present at concentrations exceeding the tolerable doses [16].

Ningxia is a typical developing region in China. Northern Ningxia is an important industrial area that is home to several industrial parks; untreated emissions have resulted in environmental pollution in this area. In recent years, researches on the contamination status of this area have been conducted [17, 18]. Groundwater and soil in this area have been polluted to some extent by multiple PTEs. For example, the soil is seriously contaminated with Cd, with concentrations exceeding the standard level; the concentrations of Zn, Pb, Cu, and Cr in the soil are higher than the background values for Ningxia [18]. However, soil samples analyzed in Ref. [18] were collected from industrial parks; the surrounding agricultural soil was not evaluated. The Yellow River is the second largest river in China and provides the drinking water, domestic water, and agricultural irrigation water for the regions along the river. However, rapid population growth and industrial development have resulted in the direct discharge of pollutants into the river, including metals, causing the water quality to deteriorate [19, 20]. For example, filtered water from the Yellow River in the Ningxia area was reported to be severely polluted by Cr [21]. Given that crops could be strongly affected by metals in soil and irrigation water, the levels of PTEs in agricultural soil and irrigation water around an industrial area should be determined. Previous studies indicated that the physicochemical properties of the soil and irrigation water, such as pH and organic matter (OM) of soil, pH and salinity (usually expressed as electrical conductivity (EC)) of water, affected metal accumulation in plants [22, 23]. Therefore, important physicochemical properties of soil and irrigation water also should be analyzed.

The consumption of food grown in local fields contaminated with PTEs presents a health risk for local inhabitants [24]. Risk assessments are performed using various indices, including the hazard quotient (HQ) [25], hazard index (HI) [26], and morbidity status (MS) [27]. Although numerous studies have been carried out in some heavily polluted regions [28] or developed areas [29] of China, less attention has been paid to developing regions such as Ningxia province. Therefore, the potential health risks of PTE contamination to local inhabitants in developing regions should be evaluated.

This study was carried out in northern Ningxia, China, where the Yellow River has been used to irrigate crops for several decades. Maize (*Zea mays* L.) is the most widely grown crop in the study area. The objectives of this study were to (1) determine the levels of six common PTEs (Cd, Pb, Cr, Zn, Cu, and As) in maize, associated soil, and irrigation water; (2) assess the metal accumulations ability of maize; (3) evaluate the hazardous health risks of PTEs exposure by maize consumption; and (4) calculate the most sensitive factors affecting health risks.

## Methods

### Study area and sample collection

The study area was located in Northern Ningxia, China, where an industrial region called “Shizuishan industrial park” operates. Approximately eight small industrial parks with more than 300 enterprises are located near this area, including metal mining, smelting and processing, petroleum, coal, and other fuel processing, electroplating, and chemical processing facilities. The surrounding soil and groundwater would be polluted by PTEs from the industrial park. The Yellow River passes through this area and water from the Yellow River is used to irrigate the surrounding agricultural fields. The population in this area is approximately 800,000. The climate belongs to the warm temperate zone and maize is the major crop.

**Fig. 1:**
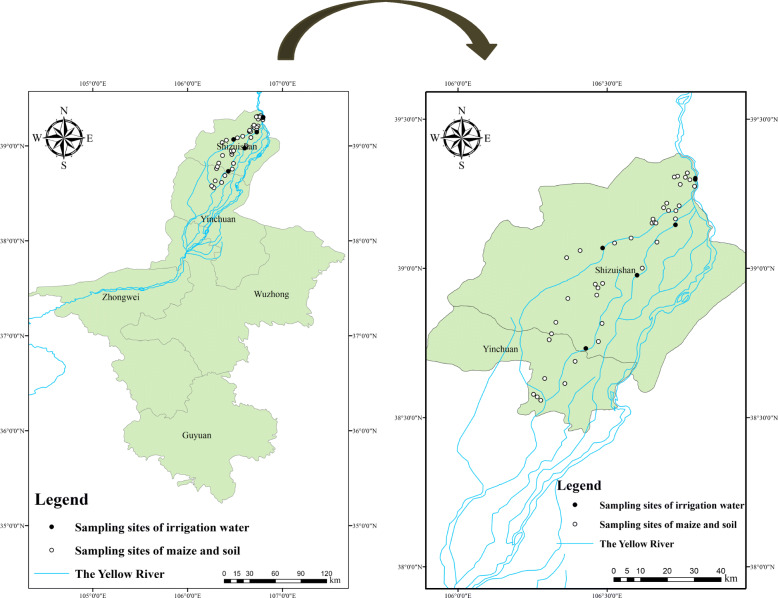
Location of the study area and distribution of the sampling sites (● and ○ represent sampling sites) which was drawn using ArcGIS Desktop 10 software with the authorization number of EFL564098460

As shown in Fig. 1, 45 fresh, raw maize samples and the associated soil were collected from the cultivated area in August 2017. The sampling plot was collected randomly in the vicinity of the industrial park and the area of each sampling plot was 200 m × 200 m. In each plot five replicate samples were taken by five-point sampling method. The same variety of maize (general maize) was selected. Surface soil (0–20 cm) was collected using a wooden spatula through five-point sampling method.

The method of irrigation water sampling was referred to the national standard of NY/T396–2000 [30]. Given that not every site has irrigation water when sampling, water sample was collected when the sampling site was near the tributary of the Yellow river. Five points were suggested by the national standard. Thus, five irrigation water samples were collected from the tributary of the Yellow river near the point of maize sampling in the present study. Each water sample was collected in a polypropylene bottle, and added with 1 mL of concentrated nitric acid (HNO_3_) to eliminate microbial activity.

The samples of maize and soil were collected by the permission of the peasant household, while irrigation water was collected without any permission. All the samples were packed, labeled and immediately transported to the laboratory.

### Chemical analysis

Maize was pre-treated according to the national standard of NY/T398–2000 [31]. The samples were dried in an oven (DHG-9030A, China) at 60 °C and powdered using a grinding mill (0.2 mm sieve). The sifted samples (0.5 g) were weighed into digestion tubes, and added with 10 mL of digestion solution (*v:v*, HNO_3_:HClO_4_ = 4:1). After cold-digestion overnight, the mixture was further digested with a block digester at 120 °C until the solution was clear. The digested samples were then diluted to a volume of 10 mL with Milli-Q water. The soil samples were prepared referring to the national standard of NY/T 395–2012 in China [32]. The soil was air-dried until reaching a constant weight and powdered using a grinding mill (0.2 mm sieve). Subsequently, 12 mL of nitric acid:hydrofluoric acid (*v:v*, HNO_3_:HF = 5:1) was added to 0.35 g of each soil sample in a Teflon digestion tube. The mixture was heated at 120 °C until the solution volume reached approximately 3 mL. The sample was then added with 5 mL of perchloric acid was added to continue the digestion until the solution was clear. The acid in the sample was evaporated at the same temperature until 1–2 mL of the solution remained. The solution was then transferred to 50 mL colorimetric tube and diluted to a final volume of 50 mL with water. Irrigation water samples were treated according to the national standard of GB 5084–2005 in China [33]. The samples were filtered with 0.45 μm filters, and 20 mL of each filtered solution was digested with 5 mL of nitric acid. The digested samples were diluted to 10 mL with Milli-Q water.

The contents of PTEs (Cd, Pb, Cr, Zn, Cu, and As) were determined by ICP-OES (Varian710-ES, USA). Quality control was performed to ensure the the accuracy and precision of the experiment procedures (Table 1S). Each analysis was performed in triplicate. In each sample batch, a blank reagent, standard reference soil (GBW07419), cabbage (GBW10014), and a multi-element standard solution (GNM-M122877–2013) were used when soil, maize and water were treated. The correlation coefficients of each element were > 0.9990. The recoveries of elements ranged from 85 to 110%, and the relative standard deviation (RSD) values were < 5%.

Some physicochemical properties of soil and irrigation water were evaluated. Soil pH and organic matter (OM) were measured the laboratory according to the standard of NY/T 395–2012 in China [32]. Water pH and electrical conductivity (EC) were detected in situ according to the standard of GB 5084–2005 in China [33].

### Questionnaires on maize consumption

To determine the maize consumption habits of residents, questionnaires were distributed in villages close to the sampling sites. Food frequency method was used to design the questionnaires in this study [34]. A total of 103 local inhabitants who consumed self-planted maize were selected to complete the questionnaire considering age distribution and gender balance. The questionnaire includes information about age, gender, body weight, frequency, and quantity of maize consumption, and maize source. The maize consumption information of the residents was used for health risk assessment. As experts suggested that the dietary structure of infants (< 3 years old) is different from that of adults, infants were not considered in the current study.

### Health risk assessment

#### Deterministic assessment

Among the six metals considered, Cd, Pb, Cr, Zn, and Cu pose non-carcinogenic health risks through oral exposure, while As poses both non-carcinogenic and carcinogenic health risks upon oral exposure. The non-carcinogenic effect of an individual metal could be evaluated by HQ value calculated using Eq. (1) [35]:

1 $$ \mathrm{HQ}=\mathrm{EXPO}/\mathrm{RfD}, $$

where EXPO is the daily exposure to metals (mg/(kg·day)), and RfD is the reference dose (mg/(kg·day)) suggested by the United States Environmental Protection Agency (USEPA) or World Health Organization (WHO). To evaluate exposure to two or more metals, HI [Eq. (2)] value was used to evaluate the total non-carcinogenic health risk [36]:

2 $$ \mathrm{HI}={\sum}_1^n{\mathrm{HQ}}_n. $$

If HQ or HI is less than 1, then no obvious non-carcinogenic risk exists. EXPO was determined using Eq. (3) [37]:

3 $$ \mathrm{EXPO}=\frac{C\times \mathrm{DI}\times \mathrm{EF}\times \mathrm{ED}}{\mathrm{BW}\times \mathrm{LT}}, $$

where C (mg/kg) represents the concentration of PTEs in maize; DI (g/day) is the daily intake of maize; EF (day/year) is the exposure frequency determined from the questionnaire; ED (year) is the exposure duration; BW (kg) is the body weight of residents determined from the questionnaire; and LT (year) is the lifetime of residents in days (presumed to be 70 years).

Carcinogenic risk (*R*) caused by As was determined using Eq. (4) [37]:

4 $$ R=\mathrm{SF}\times \mathrm{EXPO}. $$

The value of SF suggested by the USEPA is 1.5 (mg/kg/day)^− 1^ [37]. The negligible carcinogenic risk level suggested by the USEPA is 10^− 6^, while the level set by the WHO is 10^− 5^; the maximum acceptable level suggested by USEPA is 10^− 4^ [38].

#### Probabilistic assessment

Probabilistic estimation was performed to assess uncertainty and variability in risk assessment. Monte Carlo technique was used because deterministic estimation only provides the mean value of population exposure and does not accurately estimate the exposure of the population. Monte Carlo technique was performed to calculate the distribution of exposure and health risk of the population.

Probabilistic distributions of the health risk were generated by inputting the variability in exposure factors. Based on the chemical analyses and questionnaires, the distributions of parameters (like concentration of metals, daily intake of maize, exposure frequency, and body weight, and so on) were determined. The best-fitting distribution for each variable was determined by fitting a number of parametric distributions (e.g., lognormal, gamma, and Weibull). Anderson–Darling (AD) test combined with other tests was used to determine the goodness-of-fit for each distribution. The process was realized using Oracle© Crystal Ball software.

The probabilistic estimation of health risks, which was used Monte Carlo technique in Crystal Ball software, was based on the 10th, 50th, and 90th values. In the present study, Monte Carlo simulation was allowed to run for 10,000 iterations by drawing parameter values randomly from the distribution functions obtained before. The proportion of the population exceeding the acceptable health risk level was calculated.

### Sensitivity analysis

Sensitivity analysis in Crystal Ball software was performed to confirm variables that pose the greatest effect on health risk. First, the rank correlation coefficients between the exposure factors and health risk were determined using probabilistic estimation. Subsequently, the contribution of each variable was calculated by squaring the variance. Finally, the results were normalized to 100%, and the sequence of contributing variables was generated.

### Statistical methods

Mean, standard deviation (SD), minimum, maximum, and variable coefficient (C.V) were calculated in Microsoft Excel 2010 (Microsoft Ins., USA). ANOVA and correlation analysis were performed in SPSS 17.0 (IBM Ins., USA). The determination of the best-fitting distribution, Monte Carlo simulation, and sensitivity analysis were all carried out in Crystal Ball software (Oracle© Ins., USA). ArcGIS Desktop 10 (ESRI Ins., USA, Authorization number: EFL564098460) was used to map the sampling sites.

## Results

### PTEs levels in maize, associated soil, and irrigation water

Some important physicochemical properties of soil and water were shown as follows: The soil pH was 8.47 ± 0.55 and the OM was 17.92 ± 8.52 g/kg. The water pH was 8.13 ± 0.87 and the EC was 830.27 ± 50.28 μs/cm. The PTE concentrations in maize grains, associated soil, and irrigation water are presented in Table 1. The average concentrations of Cd, Pb, Cr, Zn, Cu, and As in maize grains were 0.037, 0.41, 2.36, 17.02, 1.04, and 0.17 mg/kg, respectively. The maximum allowable concentrations of the six metals in food are 0.1 (Cd), 0.2 (Pb), 1.0 (Cr), 50 (Zn), 10 (Cu) and 0.5 (As) mg/kg [39, 40]. Thus, the average concentrations of Pb and Cr exceeded the maximum allowable concentrations in food. However, the concentrations of the four remaining metals exceeded the standards in some samples. The measured metal level exceeded the limit, and the metal with the highest percentage was Cr (69%) followed by Pb (47%), As (18%), and Cd (2%). The average PTE concentrations in the soil samples were 0.14, 18.16, 37.25, 138.20, 19.61, and 14.18 mg/kg for Cd, Pb, Cr, Zn, Cu, and As, respectively, with large variation among the samples. The average concentrations of Zn and As exceeded the standards (GB15618–2018) for soils in China [41] with over-limit ratio of 18 and 4%, respectively. The background concentrations in Ningxia were 0.11 (Cd), 20.6 (Pb), 60.0 (Cr), 58.8 (Zn), 22.1 (Cu), and 11.90 (As) mg/kg [42]. Thus, the Zn level in the soil measured in this study was remarkably higher than the background value, the Cr level was lower, and the levels of the other metals were similar to the background values. Some sampling sites in this study were close to the industrial parks, which might explain the high metal concentrations found at these sites. However, source identification should be conducted to better understand the results. In the irrigation water samples, the mean concentrations of the six PTEs were 0.11 (Cd), 1.12 (Pb), 41.03 (Cr), 0.91 (Zn), 1.60 (Cu), and 3.60 (As) μg/L, which were below the limits acceptable in China (GB 5084–2005) [33]. The correlation analysis results showed the soil pH was significantly positively correlated with Zn concentration in maize (*P < 0.05*), and OM in soil was significantly negatively correlated with Pb concentration.

**Table 1:** The concentrations of PET in maize, the associated soil and the irrigation water

		Cd	Pb	Cr	Zn	Cu	As
Miaze (mg/kg) *n* = 45	mean	0.037	0.41	2.36	17.02	1.04	0.17
	SD	0.024	0.42	4.21	10.45	0.73	0.24
	mix	0.00025	ND	ND	0.84	0.0080	ND
	max	0.14	2.66	23.08	39.4	2.57	0.73
	C.V	63%	137%	184%	61%	70%	136%
	Safe limits^a^	0.1	0.2	1.0	50	10	0.5
	Over-limit ratio	2%	47%	69%	0%	0%	18%
Soil (mg/kg) n = 45	mean	0.14	18.16	37.25	138.20	19.61	14.18
	SD	119%	38.9%	27.6%	104%	96.5%	21%
	mix	ND	0.64	19.7	39.21	2.37-	9.08
	max	0.45	35.08	65.74	542.27	77.82	21.24
	C.V	119%	38.9%	27.6%	104%	96.5%	21%
	Safe limits^b^ (pH > 7.5)	0.8	240	350	300	200	20
	Over-limit ratio	0%	0%	0%	18%	0%	4%
	BF=C_maize_/C_soil_	0.26	0.023	0.063	0.12	0.053	0.12
Irrigation water (μg/L) *n* = 5	mean	0.11	1.12	41.03	0.91	1.60	3.60
	SD	0.14	2.01	28.90	0.72	0.82	1.27
	mix	ND	0.045	ND	0.095	0.74	2.40
	max	0.40	5.90	71.00	1.90	2.70	6.10
	C.V	126%	177%	70%	79%	52%	36%
	Safe limits^c^	10	200	100	2000	500	50
	Over-limit ratio	0%	0%	0%	0%	0%	0%

*ND* means not detected

^a^means allowable levels of heavy metals for corns in China by GB 2762-2017and NY 861–2004 standards;

^b^means allowable levels of heavy metals for soil in China by GB15618–2018 standard;

^c^means allowable levels of heavy metals for irrigated water in China by GB 5084–2005 standard

Bioaccumulation factor (BF) is an index used to evaluate the transfer potential of a metal from soil to plant [43]. In the present study, BF was calculated by the concentration of metal in maize relative to that in the corresponding soil. A BF value higher than 1 indicates a high level of metal accumulation in the plant, and a BF value lower than 1 denotes a low level of metal accumulation in the plant [44]. The BF values were far less than 1 for all six PTEs, indicating the low levels of metal accumulation in maize grains. The BF values of the six metals followed the following sequence: Cd > Zn ≈ As > Cr > Cu > Pb. The accumulation ability of Cd was the highest, while that of Pb was the lowest in maize. The results of correlation analysis (Table 2S) showed that the correlation between the BFs of Pb, Zn, and Cr had significant negative (*P < 0.01*) correlations with the corresponding PTEs in soil, while the BF of Cd had significant positive correlations (*P < 0.01*) with Cd in soil. Moreover, the relationships of the BFs of all PTEs and corresponding PTEs in maize were significantly positively correlated (*p < 0.01*).

### Body weight, daily consumption of maize, and exposure frequency of maize

The results for resident BW, DI, and EF are summarized in Table 2. Of the 103 local inhabitants who participated in this study, 49% were male, and 51% were female. In older to eliminate differences in size of the age population and considering the data of the present study, interval of 20 years was selected to divide the age group. Therefore, the participants were classified into four age groups: below 20 years, 20–40 years, 40–60 years, and more than 60 years old (occupying 25, 34, 26, and 15% of participants, respectively). The average BWs of all participants, male participants, and female participants were 54.60, 58.45, and 50.67 kg, respectively. Based on the reported statistics, the average BW values for adults in China are 62.7 kg for males and 54.4 kg for females [45]. The average BWs was slightly lower than the reported values possibly because children were considered in the current study.

**Table 2:** Exposure factors of the inhabitants by gender and age groups (mean ± SD)

	n (person)	Body weight (kg)	Daily intake of maize (g/day)	Exposure frequency (day/year)
All	103	54.60 ± 18.56	161.13 ± 97.16	15.50 ± 8.45
Male	50	58.45 ± 18.28	160.20 ± 96.27	14.34 ± 7.47
Female	53	50.67 ± 18.19	162.08 ± 99.06	16.67 ± 9.28
< 20	26	30.92 ± 15.37	118.46 ± 83.03	17.81 ± 8.75
20 ~ 40	35	62.55 ± 10.73	186.37 ± 95.42	14.06 ± 6.13
40 ~ 60	27	65.78 ± 7.68	181.48 ± 105.75	17.11 ± 10.11

In this study, the maize DI was slightly less for males (160.20 g/day) than for females (162.08 g/day). Among the different age groups, DI was the highest (186.37 g/day) in the 20–40 age group followed by the 40–60 (181.48 g/day) and > 60 (136.36 g/day) groups. The < 20 group had the lowest DI of 118.46 g/day. The maize EF was also lower for males (14.34 day/year) than for females (16.67 day/year). In contrast to DI, EF was the highest in the < 20 age group (17.81 day/year) followed by the 40–60 (17.11 day/year) and 20–40 (14.06 day/year) age groups. The > 60 group had the lowest EF of 10.36 day/year. BW, DI, and EF affected health risk, and their degrees of influence were evaluated by sensitivity analysis.

### Human health risk assessment

#### Deterministic assessment

The oral RfD values were established as 1, 1500, 300, 40, and 0.3 μg/kg/day for Cd, Cr, Zn, Cu, and As, respectively [17]. The RfD value for Pb was 3.57 μg/kg/day according to the provisional tolerable weekly intake level (25 μg/kg/week) [46] set by the WHO.

The results of the deterministic estimation of health risk are shown in Table 3. The HQ values indicate that no individual metal posed a significant non-carcinogenic risk (HQ < 1). For all inhabitants, the non-carcinogenic health risk posed by the different metals decreased in the following order: As > Pb > Zn > Cd > Cu > Cr. The combined non-carcinogenic health risk HI values were also less than 1, indicating that maize consumption was not associated to have an obvious non-carcinogenic health risk due to PTEs. The HI values for females were greater than those for males, implying that females experienced more potential non-carcinogenic health risks from PTEs than males. This finding can be attributed to the higher DI and EF but lower BW of females compared with males. Among the different age groups, the HI decreased in the following order: below 20 years > 40–60 years > 20–40 years > more than 60 years. Similarly, one study indicated that children are at a greater health risk than adults from the consumption of an individual metal in maize [47].

**Table 3:** Non-carcinogenic risk HQ, HI value of heavy metals and carcinogenic risk R value of As due to consumption of maize by deterministic estimation method

	Non-carcinogenic risk (HQ)							Carcinogenic risk (R)
	Cd	Pb	Cr	Zn	Cu	As	HI	As
All	0.0048	0.0109	0.0002	0.0071	0.0033	0.0725	0.0986	3.261E-05
Male	0.0041	0.0093	0.0002	0.0061	0.0028	0.0623	0.0848	2.804E-05
Female	0.0055	0.0127	0.0002	0.0083	0.0038	0.0845	0.1150	3.802E-05
< 20	0.0071	0.0162	0.0003	0.0106	0.0049	0.1081	0.1472	4.864E-05
20 ~ 40	0.0044	0.0100	0.0002	0.0065	0.0030	0.0664	0.0904	2.987E-05
40 ~ 60	0.0049	0.0112	0.0002	0.0073	0.0034	0.0748	0.1018	3.366E-05
> 60	0.0025	0.0057	0.0001	0.0037	0.0017	0.0378	0.0514	1.700E-05

In terms of carcinogenic risk caused by As, the average *R* value for all participants was 3.261 × 10^− 5^, which was lower than the maximum acceptable carcinogenic level set by the USEPA (10^− 4^) but higher than the negligible risk levels set by the USEPA (10^− 6^) and WHO (10^− 5^). Among the age groups, the *R* values decreased in the same order as the HI values: below 20 years > 40–60 years > 20–40 years > more than 60 years. In addition, the ANOVA results showed no significant difference in health risk between males and females (*P* = 0.137, *P* > 0.05). For different age groups, the health risk of age group of the below 20 years group was significantly higher than that of the more than 60 years group (*P* = 0.024, *P* < 0.05).

#### Probabilistic assessment

The best-fitting distributions of exposure factors were determined using Crystal Ball software. The concentrations of the metals in maize were fitted to lognormal distributions, except for the Cu concentration, which was fitted to a beta distribution. All DI, EF, and BW values were fitted to lognormal distributions, except for the BWs of males (Poisson) and females (negative binomial).

**Fig. 2:**
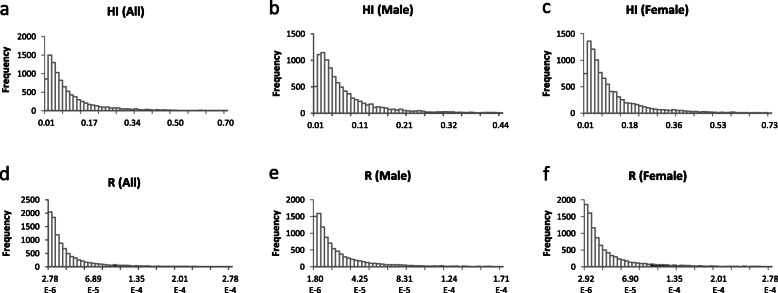
Probability exceeding 1 of HI (**a**: all inhabitants, **b**: male, **c**: female) and 10^−4^ of R (**d**: all inhabitants, **e**: male, **f**: female). The black area represents the exceeding probabilities, which were 0.62, 0.22, 1.17, 8.23, 5.12 and 10.46% for **a**, **b**, **c**, **d**, **e** and **f**, respectively

**Fig. 3:**
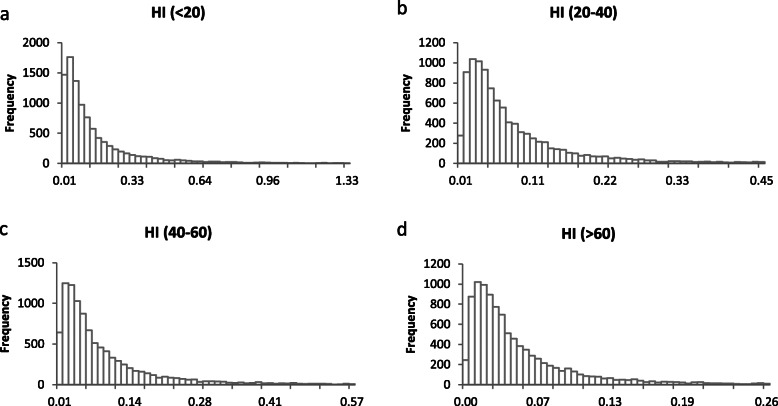
Probability exceeding 1 of HI (**a**: below 20 years, **b**: 20–40 years, **c**: 40–60 years, **d**: more than 60 years). About 2.07, 0.28, 0.53 and 0.04% of inhabitants had HI values greater than 1 for **a**, **b**, **c**, and **d**, respectively

The results of the probabilistic estimation of health risk are summarized in Table 4. For non- carcinogenic risk, all HI values were fitted to lognormal distributions. The 10th, 50th, and 90th percentile HI values were 0.02, 0.06, and 0.25 for all inhabitants; 0.01, 0.05, and 0.18 for males; and 0.02, 0.07, and 0.29 for females, respectively. Approximately 0.62% (all inhabitants; Fig. 2a), 0.22% (male inhabitants; Fig. 2b), and 1.17% (female inhabitants; Fig. 2c) had HI values greater than 1, indicating slight non-carcinogenic risk. Among the age groups, the 90th percentile HI value was the highest (0.40) for inhabitants aged below 20 years and the lowest for those aged more than 60 years (0.11). Approximately 2.07% (under 20 years old), 0.28% (20–40), 0.53% (40–60), and 0.04% (over 60 years old) of inhabitants had HI values greater than 1 (Fig. 3), indicating very low non-carcinogenic risk in all age groups. Basing on the HI values determined via probabilistic estimation, we can conclude that the non-carcinogenic health risks resulting from the studied metals are not significant.

**Table 4:** The statistics of probabilistic estimation of HI and R values

	Non- carcinogenic risk (HI)					Carcinogenic risk(R)(×10^−5^)				
	Distribution	Parameters	10%	50%	90%	Distribution	Parameters	10%	50%	90%
All	Lognormal	Location:0.00, Mean:0.11, SD:0.18	0.02	0.06	0.25	Lognormal	Location:0.00, Mean: 3.8, SD: 8.3	0.29	1.61	8.62
Male	Lognormal	Location:0.01, Mean:0.08, SD:0.11	0.01	0.05	0.18	Lognormal	Location:0.00, Mean:4.4, SD: 9.5	0.26	1.28	6.35
Female	Lognormal	Location:0.00, Mean:0.13, SD:0.20	0.02	0.07	0.29	Lognormal	Location:0.00, Mean: 3.3, SD: 2.0	0.34	1.85	10.03
< 20	Lognormal	Location:0.00, Mean:0.18, SD:0.29	0.02	0.09	0.40	Lognormal	Location:0.00, Mean: 2.5, SD: 3.7	0.40	2.31	13.40
20 ~ 40	Lognormal	Location:0.00, Mean:0.09, SD:0.12	0.02	0.06	0.20	Lognormal	Location:0.00, Mean: 3.1, SD: 5.7	0.32	1.51	7.11
40 ~ 60	Lognormal	Location:0.00, Mean:0.10, SD:0.14	0.02	0.06	0.22	Lognormal	Location:0.00, Mean: 3.4, SD: 6.9	0.30	1.54	7.70
> 60	Lognormal	Location:0.00, Mean:0.05, SD:0.06	0.01	0.03	0.11	Lognormal	Location:0.00, Mean: 1.8 SD: 3.2	0.19	0.88	4.07

**Fig. 4:**
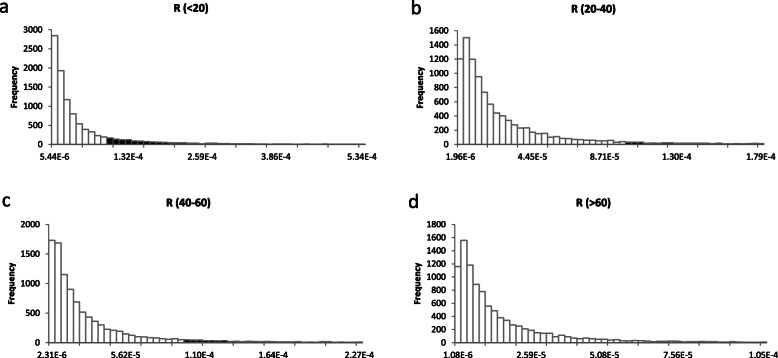
Probability exceeding 10^− 4^ of R (**a**: below 20 years, **b**: 20–40 years, **c**: 40–60 years, **d**: more than 60 years), the black area represents the exceeding probabilities, and about 14.81, 7.18, 5.83 and 2.08% for **a**, **b**, **c**, and **d**, respectively

To evaluate carcinogenic risk, all *R* values were also fitted to lognormal distributions. The 10th, 50th, and 90th values of *R* for all inhabitants were 0.29 × 10^− 5^, 1.61 × 10^− 5^, and 8.62 × 10^− 5^, respectively. The value of *R* exceeded the maximum acceptable level of 1 × 10^− 4^ (USEPA) in 8.23% of inhabitants (Fig. 2d), while *R* was greater than the negligible level of 1 × 10^− 5^ (WHO) for approximately 64.26% of inhabitants (Fig. 1Sa). The 10th, 50th, and 90th values of *R* were 0.26 × 10^− 5^, 1.28 × 10^− 5^, and 6.35 × 10^− 5^ for males and 0.34 × 10^− 5^, 1.85 × 10^− 5^, and 10.03 × 10^− 5^ for females, respectively. Approximately 5.12% of male inhabitants (Fig. 2e) and 10.46% of female inhabitants (Fig. 2f) had *R* values greater than 1 × 10^− 4^, while 58.28% of male inhabitants and 68.30% of female inhabitants had *R* values greater than 1 × 10^− 5^ (Fig. 1Sb and Fig. 1Sc); thus, the carcinogenic risk was greater for females than for males. The respective 10th, 50th, and 90th values of *R* were 0.40 × 10^− 5^, 2.31 × 10^− 5^, and 13.40 × 10^− 5^ for inhabitants aged below 20 years; 0.32 × 10^− 5^, 1.51 × 10^− 5^, and 7.11 × 10^− 5^ for inhabitants aged 20–40 years; 0.30 × 10^− 5^, 1.54 × 10^− 5^, and 7.70 × 10^− 5^ for inhabitants aged 40–60 years; and 0.19 × 10^− 5^, 0.88 × 10^− 5^, and 4.07 × 10^− 5^ for inhabitants aged more than 60 years old. Approximately 14.81% (under 20 years old), 7.18% (20–40), 5.83% (40–60), and 2.08% (over 60 years old) of inhabitants had *R* values greater than 1 × 10^− 4^ (Fig. 4), while 73.24% (under 20 years old), 63.26% (20–40), 63.47% (40–60), and 45.74% (over 60 years old) of inhabitants had *R* values greater than 1 × 10^− 5^ (Fig. 2S). Thus, the carcinogenic risk differed among the age groups.

### Sensitivity results

The sensitivity analyses showed that the contributions of As concentration, maize DI, maize EF, and Pb concentration to non-carcinogenic risk were 35.8, 29.4, 20.7, and 1.8%, respectively. The contributions of As concentration, maize DI, and maize EF to carcinogenic risk were 61.0, 18.5, and 13.1%, respectively. These results imply that controlling the concentration of As would effectively reduce the health risk for local inhabitants.

## Discussion

### Levels of PTEs and BF in maize

The PTE concentration in samples of maize irrigated by waste water were significantly higher (*P* < 0.05) than that irrigated by unpolluted water [48]. The PTE levels of Cd, Pb, Cr, Zn, Cu, and As in maize growing near a petrochemical industry complex were 0.0442, 0.0595, 0.2996, 20.42, 4.49, and 0.0428 mg/kg, respectively [49], and the levels of Pb, Cr, Cu, and As were lower than the present results; hence, maize in the current study was more contaminated. Maize in another industrial area was found to have Pb, Cd, Zn, and Cu levels of were 0.129, 0.021, 3.4, and 2.6 mg/kg, respectively [50], which were below the standard values and our study values. Although their study area was an industrial area, the maize samples were collected from a market and may not have been grown near an industrial zone.

Previous study found that maize grains have lower BF values compared with other parts [51], indicating the low accumulation ability of PTEs in maize grains. Researchers also found that Cd is the easiest transferred metal, and Pb is the hardest transferred metal in maize [51, 52]. Wang et al. [53] collected data from many previous studies and fitted the accumulation equations of PTEs in maize grains. A parameter in the accumulation equation that reflected accumulation ability for Cd was the highest, while that for Pb was the lowest. Their results could prove findings of the present study. The BFs of PTEs in some other plants were consistent with maize. Zhuang et al. [54] found that the BF of Pb in soybeans was less than that of other PTEs. Wang et al. [53] reported Cd in wheat grains was more mobile than other PTEs and the BF of PTEs was also significantly correlated with corresponding PTEs in soil. Moreover, The BFs were related to plant species, heavy metal type, and the surrounding environment. In soil-plant system, PTE accumulations in plant are mainly dependent on metal activity and are greatly influenced by physicochemical properties of soil [55]. For example, high pH makes soil electronegative, thereby increasing the mobility of heavy metals and facilitating the metal adsorption by plants [56]. Organic matter in soil easily combines with metal ions and form organic complexes, which can reduce the availability, mobility, and accumulation of metals [57]. The present results were consistent with the explanation.

The PTE contents in irrigation water were very similar to a previous report, which indicated PTEs concentrations in their filtered water samples of Cd (ND–0.11 μg/L), Cr (74.80–94.70 μg/L), Cu (0.68–2.79 μg/L), Pb (ND–0.82 μg/L), and Zn (0.19–1.82 μg/L) [21]. However, the above samples along with the water samples in the present study were filtered and did not include suspended particles or sediment. According to another study, the PTE concentrations in suspended particulate matter of the Yellow River were 0.428 mg/kg for Cd, 74.9 mg/kg for Cr, 40.1 mg/kg for Cu, 32.6 mg/kg for Pb, and 13.6 mg/kg for As [58], which were below the standards set for soils but higher than the background values in local soil. Moreover, the PTEs concentrations in suspended particles and sediment from the Yellow River [21] were as follows: Cd (0.23–1.09 mg/kg), Cr (64.50–84.90 mg/kg), Cu (25.40–42.20 mg/kg), Pb (20.80–31.70 mg/kg), and Zn (72.50–107.00 mg/kg) for suspended particles and Cd (0.23–1.09 mg/kg), Cr (64.50–84.90 mg/kg), Cu (25.40–42.20 mg/kg), Pb (20.80–31.70 mg/kg), and Zn (72.50–107.00 mg/kg) for sediments. These results imply that PTEs in the Yellow River primarily accumulate in suspended particles and sediment, which should receive more attention.

### Health risk assessment

Non-carcinogenic risk caused by PTEs through maize consumption was not obvious in other reports, similar to the present study. For the contribution of different PTEs to non-carcinogenic risk through maize consumption, the order of this study was As > Pb > Zn > Cd > Cu > Cr. Hu et al. [52] also found As was the major contributor to health risk. El-Hassanin et al. [47] found that Pb showed higher level of health risk index than Cd. However, Yu et al. [59] found Zn and Cu were the most important contributors to health risk (> 80%). Carcinogenic risk caused by As through maize consumption was seldom studied. Most reports have focused on carcinogenic risk resulting from rice consumption [60–62], Jiang et al. [63] found that among different food sources, rice has the greatest contribution to total As daily intake.

In the present study, the estimates of health risk from PTE exposure are only for maize consumption and do not account for any other sources. In reality, health risk is affected by various sources of PTEs such as different foodstuffs (e.g., rice, wheat, and vegetables) and drinking water. Cai et al. [64] found rice consumption was the major source of PTEs exposure which accounting for more than 70% of the total HQs. The intake of vegetables was a second important source with approximately 10% of the total HQs. Cao et al. [26] also observed rice consumption was the major contributor to HI, and vegetable intake was the next greatest contributor.

Other pathways of PTE exposure including dust inhalation and dermal contact also contribute to health risks. Hu et al. [52] calculated health risk under three pathways (ingestion, dermal contact, and inhalation) of exposure to heavy metals in soil and found that ingestion was the main pathways associated with health risk. Sawut et al. [65] found the ingestion and/or dermal contact of soil carcinogenic risk lead by As was higher than the acceptable risk level. Therefore, exposure from other sources should be considered to estimate the total risk in future works.

### The uncertainties of risk assessment

Health risk assessment of PTEs through food consumption in previous reports was mainly assessed by deterministic estimation; however, deterministic assessment may overestimate or underestimate risks because of the uncertainty of metal concentration and the variability of exposure parameters. Probabilistic estimation is introduced to provide additional useful information. The proportion of residents exceeding RfDs could be obtained, and correlations between different variables and risk results could be provided. Cao et al. [26] evaluated potential health risks using probabilistic estimation and found that 0.00,4.18, 0.36, 0.03, 0.00 and 0.00% of inhabitants exceeded the corresponding RfD for Cr, Cu, Zn, Cd, Hg and Pb, respectively. Hang et al. [64] found that 3.9, 1.9, and 0.6% of inhabitants exceeded the corresponding RfD for Cu, Pb, and Cd, respectively. Jiang et al. [60] found the proportion of inhabitants exceeding the RfD of As was 27.85% and daily intake of rice, metal concentration in rice and body weight were most relevant variables to health risk results. These previous studies only focused on element exposure and paid less attention to different populations. In the present study, we calculated the proportion exceeding the threshold value for HI and R in different gender and age groups. The corresponding results would provide suggestion to different populations to consume maize.

## Conclusion

Although the average PTE concentrations in soil and irrigation water did not exceed the national standards, the average contents of two metals (Pb and Cr) in maize exceeded the standards for food. The BF values of the six metals in maize were all at low levels and the accumulation ability followed the order of Cd > Zn = As > Cr > Cu > Pb. The non-carcinogenic health risks to inhabitants was not obvious (HI < 1), while the carcinogenic risk from As exposure was lower than the maximum acceptable level and higher than the negligible level. Females are at greater risk than males, but the difference was not significant. Health risks were the highest in the age group of below 20 years. Among different exposure factors, arsenic level and daily intake of maize were the most sensitive factors that affect the health risk results.

## Supplementary Information

**Additional file 1:** **Table S1.** The results of quality control in maize, the associated soil and the irrigation water. **Table S2.** Correlations between BF and levels of PTEs in soil and maize. **Fig. S1.** Probability exceeding 10^− 5^ of R (a: all inhabitant, b: male c: female), the black area represents the exceeding probabilities, and about 64.26, 58.28 and 68.30% for a, b, c, and d, respectively. **Fig. S2.** Probability exceeding 10^− 5^ of R (a: below 20 years, b: 20 ~ 40 years, c: 40 ~ 60 years, d: more than 60 years), the black area represents the exceeding probabilities, and about 73.24, 63.26, 63.47 and 45.74% for a, b, c, and d, respectively.

## Data Availability

The datasets used and/or analysed during the current study available from the corresponding author on reasonable request.

## Abbreviations

BW: body weight
C: concentration of elements
DI: daily intake
ED: exposure duration
EF: exposure frequency
EXPO: exposure to metals
HQ: hazard quotient
HI: hazard index
MS: morbidity status
ICP-AES: inductively coupled plasma- optical emission spectrometry
I.Q.: Intelligence Quotient
PTEs: potentially toxic elements
R: carcinogenic risk of As
RfD: reference dose
SF: slope factor
SD: standard deviation
BF: Bioaccumulation factor
USEPA: United States Environmental Protection Agency
WHO: World Health Organization
