# Supplementary material for: Potentially toxic element (PTE) levels in maize, soil, and irrigation water and health risks through maize consumption in northern Ningxia, China
Source: BMC Public Health. 2020 Nov 16;20:1729. doi: 10.1186/s12889-020-09845-5 (PMC7670719; doi:10.1186/s12889-020-09845-5)
Supplement: Supplementary file 1 — Additional file 1: Table S1. The results of quality control in maize, the associated soil and the irrigation water. Table S2. Correlations between BF and levels of PTEs in soil and maize. Fig. S1. Probability exceeding 10− 5 of R (a: all inhabitant, b: male c: female), the black area represents the exceeding probabilities, and about 64.26, 58.28 and 68.30% for a, b, c, and d, respectively. Fig. S2. Probability exceeding 10− 5 of R (a: below 20 years, b: 20 ~ 40 years, c: 40 ~ 60 years, d: more than 60 years), the black area represents the exceeding probabilities, and about 73.24, 63.26, 63.47 and 45.74% for a, b, c, and d, respectively. [file 12889_2020_9845_MOESM1_ESM.docx]

Table 1S The results of quality control in maize, the associated soil and the irrigation water.

|  |  | Correlation coefficients(R^2^) | Recovery (%) | RSD (%) | Certified reference  (mg/kg) | Certified value  (mg/kg) |
| --- | --- | --- | --- | --- | --- | --- |
| Miaze | Cd | 0.9999 | 110 | 3.9 | 0.033 | 0.035±0.006 |
|  | Pb | 0.9991 | 101 | 4.5 | 0.16 | 0.19 ± 0.03 |
|  | Cr | 0.9999 | 98 | 1.9 | 2.0 | 1.8 ± 0.3 |
|  | Zn | 0.9992 | 97 | 2.9 | 24 | 26 ± 2 |
|  | Cu | 0.9999 | 91 | 1.7 | 2.4 | 2.7 ± 0.2 |
|  | As | 0.9992 | 85 | 4.2 | 0.059 | 0.062 ±0.014 |
| Soil | Cd | 0.9999 | 98 | 4.5 | 0.21 | 0.22 |
|  | Pb | 0.9991 | 95 | 3.7 | 18 | 21 ± 5 |
|  | Cr | 0.9999 | 105 | 3.8 | 61 | 61 ± 5 |
|  | Zn | 0.9992 | 93 | 4.5 | 55 | 51 ± 6 |
|  | Cu | 0.9999 | 92 | 2.9 | 18 | 17 ± 1 |
|  | As | 0.9992 | 87 | 4.8 | 10.2 | 9.8 ± 0.9 |
| Irrigation | Cd | 0.9999 | 91 | 1.2 | 100 μg/L | 100 μg/L |
| water | Pb | 0.9991 | 94 | 1.5 | 99 μg/L |  |
|  | Cr | 0.9999 | 99 | 1.6 | 101 μg/L |  |
|  | Zn | 0.9992 | 95 | 1.2 | 100 μg/L |  |
|  | Cu | 0.9999 | 102 | 1.9 | 100 μg/L |  |
|  | As | 0.9992 | 98 | 2.4 | 99 μg/L |  |

Table 2S Correlations between BF and levels of PTEs in soil and maize.

|  | BF-Cd | BF-Pb | BF-Cu | BF-Zn | BF-Cr | BF-As |
| --- | --- | --- | --- | --- | --- | --- |
| Cd-soil | **.402**** | -.267 | -.087 | .065 | .100 | .131 |
| Pb-soil | .157 | -**.509**** | .018 | .201 | .241 | .046 |
| Cu-soil | **.306*** | .034 | .103 | .124 | .065 | -.030 |
| Zn-soil | -.150 | .137 | -.028 | **-.724**** | **-.425**** | .152 |
| Cr-soil | -.129 | .202 | -.037 | **-.576**** | **-.537**** | .104 |
| As-soil | -.024 | -.053 | **-.373*** | **-.364*** | -.220 | -.079 |
| Cd-maize | **.492**** | **.301*** | .097 | .272 | .159 | .063 |
| Pb-maize | .079 | **.623**** | .030 | .159 | .091 | -.144 |
| Cu-maize | -.021 | -.014 | **.983**** | .082 | .076 | .098 |
| Zn-maize | .238 | .021 | .258 | **.740**** | **.467**** | .102 |
| Cr-maize | .260 | .153 | **.501**** | **.455**** | **.437**** | -.106 |
| As-maize | .201 | -.160 | -.023 | -.202 | -.225 | **.966**** |

***P* < 0.01.

* *P* < 0.05.

**Fig.1S** Probability exceeding 10^-5^ of R (a: all inhabitant, b: male c: female), the black area represents the exceeding probabilities, and about 64.26%, 58.28% and 68.30% for a, b, c, and d, respectively.

**Fig.2S** Probability exceeding 10^-5^ of R (a: below 20 years, b: 20~40 years, c: 40~60 years, d: more than 60 years), the black area represents the exceeding probabilities, and about 73.24%, 63.26%, 63.47% and 45.74% for a, b, c, and d, respectively.
